# Research on the cultivation of sustainable development ability of higher vocational students by creative thinking teaching method

**DOI:** 10.3389/fpsyg.2022.979913

**Published:** 2022-10-05

**Authors:** Xin-Zhu Li, Chun-Ching Chen, Xin Kang

**Affiliations:** ^1^College of Design, National Taipei University of Technology, Taipei, Taiwan; ^2^Department of Interaction Design, National Taipei University of Technology, Taipei, Taiwan; ^3^School of Design, NingboTech University, Ningbo, China

**Keywords:** creative thinking teaching, learning outcomes, material creation course, vocational education, learning assessment

## Abstract

Education for sustainable development (ESD) is an important guideline for United Nations Sustainable Development Goals. Students' creative thinking can be applied to various disciplines, promoting sustainable learning. Most of Taiwan's beauty and hairdressing technical education teachers mainly teach students to imitate, and students' works lack creativity and thinking. A total of 43 higher vocational college students participated, 23 of whom were in the experimental group using the creative thinking teaching method and 20 of whom were in the control group using the traditional teaching method. The results show that the creative thinking teaching method can effectively improve students' learning outcomes in the multimedia material creation course, including breaking through the limitation of thinking, putting forward different ideas and answers, and constantly innovating, to make the presented results more creative and meaningful. The creative thinking teaching methods solve students' trouble in creative problem solving, enhance students' problem solving and critical thinking skills, and improve students' involvement in the study.

## Introduction

Sustainability has become an increasingly important topic. Education for sustainable development (ESD) is regarded as a key driving force for United Nations Sustainable Development Goals (SDG) (Rieckmann, 2017; Leicht et al., 2018; Zhou and Lee, 2022). Higher education has regarded sustainable development as an important education goal (Molderez and Ceulemans, 2018; Sidiropoulos, 2022). Especially, creative thinking is an essential and important part of modern sustainable education (Ocetkiewicz, 2021), but is underestimated in many formal educations, especially technical and vocational education.

**Figure 1:**
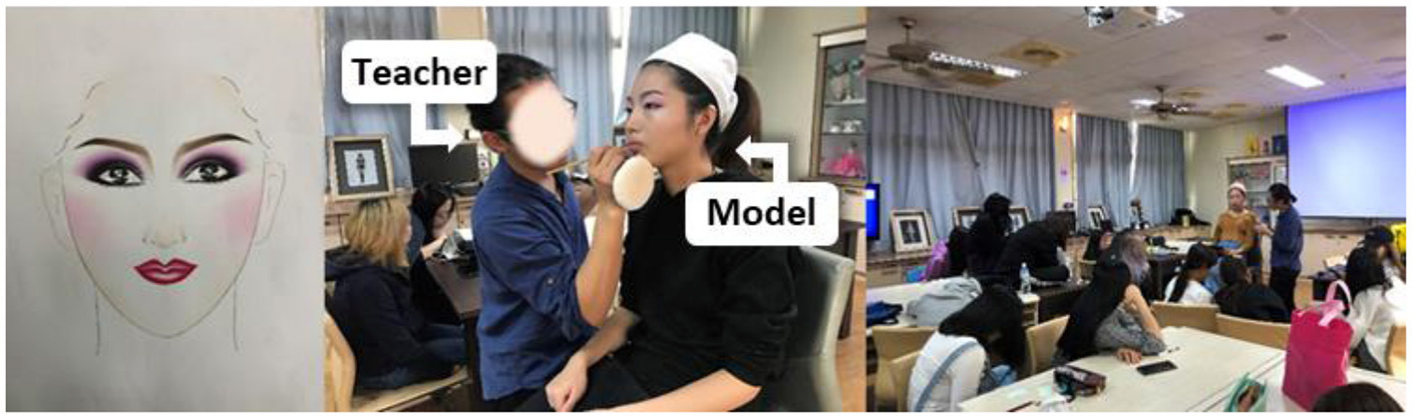
Traditional teaching process of cosmetology teacher.

Beauty and hairdressing is an important part of technical and vocational education. The training goal is to enable students to have professional knowledge and practical skills related to hairdressing and cultivate talents for the industry (Chen and Liu, 2019). Most of the Department of beauty and hairdressing courses are based on traditional teaching, which is combined with the content of the Vocational Training Bureau's skill test and lack of space to provide students with diversified thinking (Wu et al., 2014). In teaching, the teacher will first demonstrate and then let the students imitate, strengthen the students' professional ability in many exercises, and make works by observing, imitating, and then observing. When students imitate the works demonstrated by teachers and repeat practice during the course (Hsia et al., 2021), they are often limited by the standardized template framework, such as eye-brow angle, color, the position of blush, etc. The traditional teacher-centered teaching methods limit students' creative thinking ability and reduce their interest in learning. This is the current dilemma of beauty and hairdressing technical education, as shown in Figure 1. Therefore, beauty and hairdressing technical education in higher vocational colleges need to try to improve the current dilemma through different teaching methods.

Many studies have pointed out that student-centered creative thinking teaching method can enhance students' creative thinking ability (Guilford, 1968; Torrance, 1972; Chou, 2004, 2013; Chen, 2006; Black et al., 2015). It is suggested that educators use non-traditional teaching strategies to interact with students to arouse students' interest in learning. Creative thinking skills can be integrated into various disciplines, and can also be used in daily life (Lai, 2006), such as music education courses (Hickey and Webster, 2001; Burnard, 2012; Kladder and Lee, 2019), fashion design courses (Black et al., 2015), English drama courses (Dora To et al., 2011), nursing education (Ku and Kuo, 2016), etc. It can be seen that the creative thinking teaching method can be applied in various disciplines, but there is a lack of application of creative thinking teaching in Taiwan's higher vocational education.

The implementation principle of creative thinking teaching is to continuously use various creative thinking strategies in the process of teaching, let students put forward multiple opinions, do not criticize students' ideas, and encourage autonomous learning and provide attempts (Morris, 2006; Lin, 2008; Huang, 2011). The positive interaction between teachers and students can effectively improve learning outcomes (Huang, 2011). Chen et al. (2022) results show that the students' imagination was significantly improved by being involved in the creative learning activities. Creative thinking skills in the process of teaching can effectively stimulate students' creativity, increase the interaction between teachers and students, and effectively enhance students' participation. There is reasonable evidence to suggest that providing diverse appropriate materials, tools, and other resources can stimulate students' creativity (Kennedy, 2010; Robson and Jaaniste, 2010; Davies et al., 2013). Through the above literature, we can find that the creative thinking teaching method is a student-centered teaching method that can effectively enhance students' creative thinking skills, learning motivation, and learning outcomes through different teaching strategies and materials.

Creativity and creative thinking courses have become the future development trend. Creative thinking is considered to be an important ability in the 21st century (Fadel and Trilling, 2010), creative thinking has become a necessary ability in the future and has been paid more and more attention. Creative thinking teaching changes the traditional teaching method, integrates diversified materials, enables students to put forward different questions through thinking, and then uses diversified materials to assist the practice of creation, which is more conducive to improving students' creative thinking ability.

Although there are lots of researches on creative thinking teaching, seldom are about beauty and hairdressing technical education. Therefore, this study will make up for this literature gap, provide empirical experience for the research of related disciplines, and also hope to arouse the reflection of vocational education teachers on the role and status of teachers in thinking teaching.

This study integrates the teaching method of creative thinking into the course of material creation, and explores the comparative study on the learning outcomes of the creative thinking teaching method and the traditional teaching method.

## Literature review

### Comparison between traditional teaching method and creative thinking teaching method

Traditional teaching methods and creative thinking teaching methods are different in idea, goal, mode, method, material resources, materials, aids, and evaluation strategies. The traditional teaching method is a teacher-centered narrative teaching method, in which teachers lead all teaching activities, instill knowledge into students, emphasize recitation of knowledge and facts, and treat students as passive receivers (Mitchell and Martin, 1997); Creative thinking teaching method is a student-centered teaching method, which emphasizes students as participants and masters, and uses creative thinking strategies to teach students to solve problems and creative thinking methods, to find inspiration and stimulate creativity from reasoning (Chen, 2006; Liu et al., 2007).

In the traditional teaching method, teachers control the teaching process, and the course limits students' creativity, thus students cannot bring their ideas into play through one-way teaching (Chen, 2006). Especially in the course of cosmetology, students copy the works demonstrated by teachers just like photocopiers. In contrast, the creative thinking teaching method aims to develop learner autonomy and independence (Jones, 2007). The teacher plays the role of facilitator rather than an instructor. The teacher's goal in the learning process is to guide students to new interpretations of the learning material, thereby 'experiencing' content, As Rogers' notion that “important learning is acquired through practice.” (Kraft, 1978). In the creative thinking teaching methods, students are the focus. Therefore, it can improve students' problem-solving skills and develop their creative thinking and critical ability (Su et al., 2017). In this way, students can generate ideas, put forward hypotheses, and apply their imagination to find alternative creative ideas (Tan et al., 2016). However, creative thinking teaching methods combine various teaching strategies and show that students play a major role in the study. The literature found that pedagogical practice is one of the key environmental features in shaping student creativity (Davies et al., 2013).

### Creative thinking teaching method and learning outcomes

The teaching of creative thinking is that teachers use a variety of teaching contents, methods, and activities to cultivate students' creativity (Ku and Kuo, 2016). The creative thinking teaching method has four characteristics: (1) encourage the use of imagination, increase students' creative ability; (2) In learning activities, students are the main body, teachers give guidance in teaching, and do not monopolize the whole teaching time; (3) The learning environment should be free, safe and harmonious; (4) Teaching methods should focus on stimulating students' interest in learning, encouraging students to express their own ideas and accept different opinions and thinking, and not rush to make judgments (Lin, 1974; Cropley, 1997; Chen, 2006; Davies et al., 2013; Schweisfurth and Elliott, 2019).

In the implementation of creative thinking teaching, students often show adventurous, challenging, curiosity and imagination, and these characteristics are included in the emotional level, which are related to students' attitude, value, appreciation and motivation, and are called creative tendency (Chen, 2006). Thomas and Chan believed that creative tendency is one of the commonly used tools to measure teenagers' creativity, including risk-taking, curiosity, imagination and challenge (Thomas and Chan, 2013), which can reflect psychological state (Shi et al., 2013).

Cosmetology is a part of vocational education, and also belongs to the field of art and design. Besides training students' skills, the most important thing is to enhance their creativity. Some studies have shown that the use of multi-media materials and a wide range of resources and tools in the course can effectively enhance students' creativity (Beghetto and Kaufman, 2014; Al-Dababneh et al., 2019). Creative thinking teaching provides an environment for students to constantly think and create, and stimulates students' creativity. In the course of multi-media creation, students are guided to learn through concept establishment and technical guidance.

### Learning outcomes assessments

Learning outcomes refers to the changes in learners' knowledge, skills, and attitudes after the end of the teaching activity. Wager believes that learning outcomes are the learning goal that teachers expect students to achieve (Wager, 2003). Learning outcomes can be used as the basis for evaluating students' performance, reflecting the merits and demerits of the teaching plan, enabling students to understand their own learning status, and can be used as the basis for teachers to improve teaching and students to improve learning. Brown believes that learning outcomes are the presentation of knowledge and skills learned after formal courses (Brown, 1981). Effective assessment for Learning can improve students' attention to the course and motivate students to participate in course activities (Wu et al., 2021a,b). Kirkpatrick proposed the Four-Level Training Evaluations Model to evaluate the learning outcomes of students, which are reaction level, learning level, behavior level, and result level (Kirkpatrick, 1975; Kirkpatrick and Kirkpatrick, 2006).

Kirkpatrick's four-level evaluation is the most widely used evaluation model for learning Outcomes (Lien et al., 2007; Mollahoseini and Farjad, 2012; Sidiropoulos, 2022). According to the theory of Kirkpatrick, we can know whether the teacher's goal is achieved through the teaching process and whether the students' expectations and needs for the course are met in the learning process. The level of learning satisfaction at the reaction level can affect the learning performance during training. It can be seen that it is very important to understand the students' learning satisfaction in measuring the learning outcomes. Through the evaluation, we can better understand the real benefits of students through the learning process, as well as the suggestions for teachers' curriculum and teaching methods.

## Methods

In this study, learning outcomes are defined as the change of learners' cognition, affection, and skills through the learning and understanding of knowledge and the application of skills. According to Kirkpatrick's evaluation model after learning: reaction, learning, behavior, and result, students' learning outcomes are measured. Learning satisfaction is used as the “reaction level” evaluation, the creative tendency is used to evaluate the learning level of curriculum absorption, and the expert consensus is used to measure the behavior level of students' professional ability to apply what they have learned in the course. Since the “result level” refers to the changes in people's psychology, skills and abilities, which requires a long-term observation time process (Gagné, 1985). It is difficult to set an evaluation standard and measure time. Therefore, this study will not include the evaluation and verification of the “outcome level” and only conduct a cross-sectional study.

### Research design

Based on the research objectives, this paper adopts the quasi-experimental design method of equal group post-test design, and carries out experimental workshops for the two groups, respectively using the creative thinking teaching method and the traditional teaching method. The teaching plans of both the experimental group and the control group were prepared by researchers. The subject of the course is to design a white mask with a length of 19.5 cm and a width of 10 cm. In terms of course materials, the control group used the same material package. Materials for the experimental group were selected by students in an open way according to their own creativity, as shown in Table 1.

**Table 1:** Teaching design of experimental group and control group.

**Week**		**Experimental group A**	**Control group B**
Week 1: Introduction	Teaching content	Introduction of multi-media materials and Case study	
	Teaching strategy	Didactic Teaching, Observation method.	
Week 2: Creation expression	Teaching content	Multi-media creation visual communication interpretation, understand how artists convey stories through their works	
	Teaching strategy	Brainstorming, free association	Didactic Teaching, Observation method.
Week 3: The composition of multi-media materials	Teaching content	Deconstruction and analysis of works, let students learn the composition of multi-media materials, such as collage, deconstruction, reconstruction.	Teacher demonstrates multi-media composition (collage, deconstruction, reconstruction).
	Teaching strategy	Observation method, six W's review method	Didactic Teaching, hands-on method.
Week 4: Do	Teaching content	Creation without limitation of material	Students work with the same materials
	Teaching strategy	Hands-on method	

The subjects of this study were junior students of the department of applied cosmetology in southern Taiwan. The students were randomly divided into experimental groups and control groups. Group A was the experimental group. Group A total of 23 students (male 2, female 21) received the multimedia material creation course of creative thinking teaching method. The students were coded from SA1 to SA23. Group B was the control group and received the traditional teaching method of the multimedia material creation course. a total of 20 people (male 0, female 20). The students were coded from SB1 to SB20, as shown in Table 2. The two groups received eight 45-min courses.

**Table 2:** Summary of participants.

**Variable**	**Category**	**Experimental group A**		**Control group B**	
		**Frequency**	**Percent**	**Frequency**	**Percent**
**Gender**	Male	2	8.7%	0	0%
	Female	21	91.3%	20	100%
	Total	23	100%	20	100%
**Age**	17	13	56.5%	8	40%
	18	10	43.5%	12	60%
	Total	23	100%	20	100%
**Experience in making masks**	Yes	5	21.7%	4	20%
	No	18	78.3%	16	80%
	Total	23	100%	20	100%

### Questionnaire design

The questionnaire is divided into two parts. The first part is to measure the response level (learning satisfaction) and learning level (creative tendency) of students' learning outcomes, with total 44 questions; In the second questionnaire, the teacher assessed the behavior level of students' learning outcomes (expert consensus assessment), a total of 12 questions. The Likert five-point scale was used to measure each question, (1) strongly agree; (2) agree; (3) neither agree nor disagree; (4) disagree; (5) strongly disagree.

Questionnaire 1 was distributed to the students after the course, which was used to investigate and evaluate the differences in learning outcomes between the two groups of students applying different teaching methods. Questionnaire 2 was distributed to the students after the material creation course, the students' work photographs were submitted to experts for consensus evaluation. The expert consensus scale adopts the creativity power scale in the field of art revised by Amabile in 1983 (Amabile, 1983). In the research of consensus scale, the raters are generally divided into three categories: college teachers, high school or primary school teachers, and professionals (Lai, 2006; Kousoulas, 2010; Shih, 2013; Amabile et al., 2018; Jeffries et al., 2018). In this study, experts are divided into the above three categories, including 2 teachers from colleges and universities, 2 teachers from high schools, and 2 experts from the industry. Each expert independently evaluates students' works by using the consensus rating scale.

Through the questionnaire to evaluate the impact of creative thinking teaching method on students' learning outcomes, the literature shows that the learning outcomes can be divided into reaction level (curriculum content satisfaction and teaching method satisfaction), learning level (adventure, curiosity, imagination, challenge), behavior level (creativity, aesthetic attraction, technical advantages), and result level, this is a cross-sectional study, and the resulting level is not in the scope of this study.

#### Questionnaire 1: Students' feedback

Questionnaire 1 is divided into the following two parts:

The first part is the reaction level of learning outcomes, including content satisfaction and teaching method satisfaction. The questionnaire was prepared concerning the Creativity Training Course Teaching Feedback Form (Chou, 2013), Learning Response and Learning Interest Questionnaire (Huang, 2011), Hairdressing Course Creativity Reflection Learning Response and Learning Interest Scale (Tseng, 2009), and Home Economics Course Learning Response Scale (Chan, 2004), Learning Response Questionnaires (Wu, 2002), Teaching Response Scale (Wei, 2001), and theories proposed by Kirkpatrick and Kirkpatrick (2006).

The second part is the learning level, including risk-taking, curiosity, imagination, and challenge. This part of the questionnaire was prepared by referring to the Williams Creative Tendency Scale revised by Lin and Wang (1994) and the Creative Tendency Scale revised by Hung (2011). Students' learning outcomes were assessed from four aspects: adventure, curiosity, imagination, and challenge. A total of 20 questions were asked.

#### Questionnaire 2: Teacher evaluation index

Questionnaire 2 is the behavior level, including creativity, aesthetic appeal, and technical advantages. The consensus assessment technique proposed by Amabile is developed based on appropriate social-psychological methods. It refers to that when experts in a certain field look at products or clearly observable reactions, able to evaluate creativity (Amabile, 1983; Chang, 2013; Amabile et al., 2018). According to the characteristics of this study, 12 questions of the expert consensus rating scale proposed by Amabile were selected, which were divided into four questions of creativity group (creativity, novel use of materials, novel idea, and significance of effort). Aesthetic attractiveness group four questions (aesthetic attraction, display value, preference, and pleasing shape configuration); The technical value group uses four questions (technical advantages, overall organization, expressiveness, and fineness) to evaluate student works (Amabile et al., 2018), as shown in Table 3.

**Table 3:** Expert consensus assessment (behavioral level).

**Group**	**Factor**
Creativity	Creativity
	The novel application of material
	Novel concept
	Degree of effort
Aesthetic appeal	Overall aesthetic appeal
	Value of presentation
	Degree of liking
	Pleasing shape
Technical advantages	Technical advantages
	Overall organization
	Expressivity
	Ingenious

In the expert consensus assessment section, a validity analysis was carried out in order to determine the quality of the measuring instrument. The procedure used was expert judgment, by which an assessment of each item's quality and relevance was carried out by 4 expert judges. The expert validity of the questionnaire was evaluated by 2 higher vocational teachers and 2 college professors according to the research direction and the content of the questionnaire.

There are 12 questions in which 6 experts evaluate students' work, and take the average of 6 experts to assess the behavioral aspect of learning outcomes of the two groups. The experts were coded from E1 to E6.

43 questionnaires were collected from the two groups, of which 23 were collected from group A (experimental group), and 20 from group B (control group). The questionnaire recovery rate was 100%.

### Research hypotheses

This study explores the differences in learning outcomes between creative thinking teaching and traditional teaching, based on the above relevant literature and theoretical research, we propose the following hypotheses according to the four-level evaluation model proposed by Kirkpatrick (Kirkpatrick, 1975; Kirkpatrick and Kirkpatrick, 2006), which are response level, learning level and behavior level, respectively, and the result level is presented in the research result part.

#### Reaction level

The integration of creative thinking teaching method into the curriculum of all disciplines will improve students' learning satisfaction. In terms of students' reaction level, students hold a favorable and positive attitude toward course activities, course content and teaching methods (Li et al., 2011; Hu et al., 2016; Huang et al., 2021). Therefore, the hypothesis at the reaction level of this study is as follows:

H1a: The creative thinking teaching method is positively related with the learning satisfaction in course content satisfaction.

H1b: The creative thinking teaching method is positively related with learning satisfaction in Teaching method satisfaction.

#### Learning level

Some studies have found that the creative thinking teaching method has a significant impact on all aspects of creative tendency (Chen, 2003; Hung, 2004), but there are also studies show that there is no significant impact (Liu et al., 2020). It can be seen that the creative thinking teaching method can affect students' creative thinking ability, but it is not clear whether the creative thinking teaching method can significantly improve students' creative tendency. Therefore, this study deduces the following hypotheses based on the above relevant literature:

H2a: The creative thinking teaching method is positively related with the adventure of the creative tendency.

H2b: The creative thinking teaching method is positively related with the creative tendency of curiosity.

H2c: The creative thinking teaching method is positively related with the creative tendency of imagination.

H2d: The challenge of creative thinking teaching method is positively related with creative tendency.

#### Behavior level

Expert consensus assessment is a common way to assess students' learning outcomes, which has good consistency in student assessment (Lai, 2006; Shih, 2013). Based on relevant studies, the expert consensus assessment is divided into three parts, namely creativity, technical advantage and aesthetic appeal, respectively (Shih, 2013), we propose research hypotheses from the above three aspects:

H3a: The creativity of expert consensus assessment is positively related with the creative thinking teaching method.

H3b: The aesthetic sensibility of expert consensus assessment is positively related with the creative thinking teaching method.

H3c: The technical advantage of expert consensus assessment is positively related with the creative thinking teaching method.

**Figure 2:**
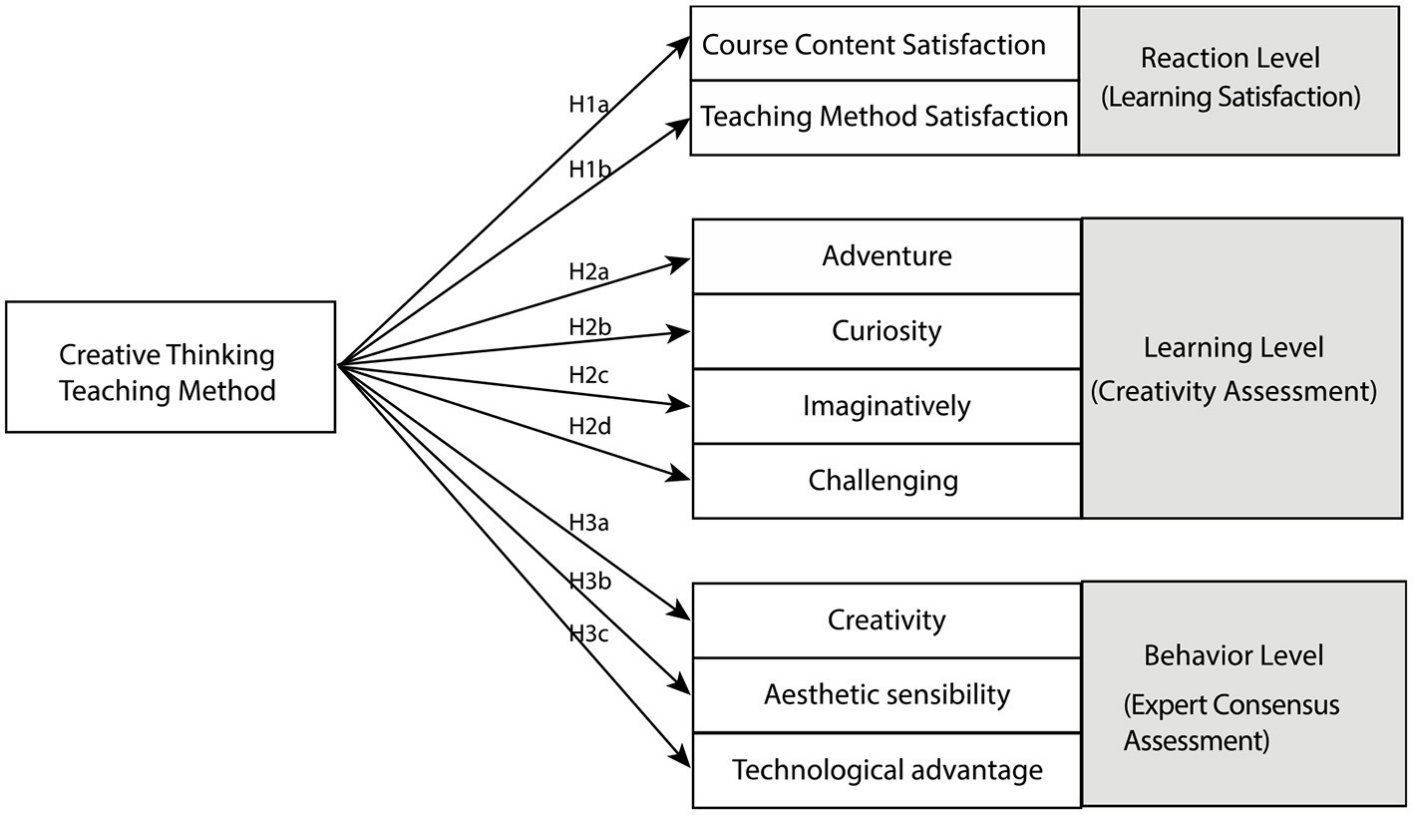
Research hypotheses.

Figure 2 shows the research hypothesis framework.

## Results

Based on the collected questionnaire data, this part analyzes the research questions and hypotheses and discusses the correlation between the creative thinking teaching method and learning outcomes. To analyze the reliability, Cronbach's Alpha was utilized. The Cronbach's Alpha value of 0.94, coefficients found obtained values higher than 0.70, and this indicates an acceptable degree of reliability between the items that shape the latent variable, as shown in Table 4.

**Table 4:** Assessment of convergent reliability.

**Items**	**Cronbach's Alpha**	
Reaction-Learning satisfaction	0.923	0.941
Learning- Creative tendency	0.925	
Behavior-Expert consensus assessment	0.985	

### Result 1: Students' feedback

According to Levene's homogeneity test of variation, whether there is a significant difference in the variation of the reaction level of learning satisfaction (satisfaction of course content and teaching method) of learning outcomes between control group B and experimental group A can be determined. Then, whether there is a significant difference in the mean value of *t*-test and significance test, and all data were analyzed using IBM SPSS 22.0.

An independent samples *t*-test was adopted to test for differences between the two groups of students about course content satisfaction and teaching method. The results are as follows: course content satisfaction (*t* = 0.403; *p* > 0.05), teaching method (*t* = 4.801; *p* = 0.000 < 0.05), indicating that there was significant difference between the creative thinking teaching method and course content satisfaction in the reaction level, and there was a significant difference between the learning satisfaction level of creative thinking teaching method and learning satisfaction, as shown in Table 5.

**Table 5:** Expert consensus assessment (behavioral level).

**Items**	**Experimental group A**			**Control group B**			***t*-value**	***p*-value**
	**Mean**	**SD**	***N***	**Mean**	**SD**	***N***		
**Learning satisfaction**	4.6993	0.37667	23	4.2417	0.44990	20	3.631	0.001**
Course content satisfaction	4.7029	0.37925	23	4.6500	0.46798	20	0.403	0.689
Teaching method satisfaction	4.6957	0.41333	23	3.8333	0.73945	20	4.801	0.000***

Mean is the average, SD is the standard derivation, and N is the number.

**p < 0.01.

***p < 0.001.

A difference analysis was conducted for experimental group A vs. control group B creative tendency. An independent samples *t*-test was adopted to test for differences between the two groups of students about adventure, curiosity, creative thinking, and challenge. The results are as follows: adventure (*t* = 1.685; *p* > 0.05), curiosity (*t* = 0.643; *p* > 0.05), creative thinking (*t* = 0.624; *p* > 0.05), challenging (*t* = 0.658; *p* > 0.05), indicating that there was no significant difference between the creative thinking teaching method and the tradition-al teaching method in the level of creative tendency (adventure, curiosity, imagination, and challenging), as shown in Table 6.

**Table 6:** Independent sample *t*-test for two groups of creativity tendency.

**Items**	**Experimental group A**			**Control group B**			***t*-value**	***p*-value**
	**Mean**	**SD**	***N***	**Mean**	**SD**	***N***		
**Creative tendency**	4.3109	0.54730	23	4.1600	0.42938	20	0.995	0.326
Adventure	4.2609	0.55411	23	3.9800	0.53469	20	1.685	0.100
Curiosity	4.4261	0.55368	23	4.3300	0.40144	20	0.643	0.524
Creative thinking	4.2696	0.60486	23	4.1600	0.53744	20	0.624	0.536
Challenging	4.2870	0.64899	23	4.1700	0.49108	20	0.658	0.514

Mean is the average, SD is the standard derivation, and N is the number.

2 weeks after the end of this experiment, two groups of students experienced the teaching method of the opposite group. In this way, all the students experienced both the traditional teaching method and the creative thinking instruction method. Then, students' feedback and what they have gained were asked. Students SA5, AS11, SA13, SA22, SB4, and SB10 from the creative thinking instruction group said, they used different thinking methods to create more narrative and creative works. Besides, students SA2, AS18, SA20, SB1, SB15 said, both of these two teaching methods could inspire their learning motivation but courses in creative thinking instruction method were more interesting. Importantly, students SA1, AS15, SB17, and SB20 said, the traditional teaching method was boring, and they preferred the creative thinking instruction method. These feedbacks were the same as what data had been presented.

### Result 2: Teacher evaluation index

A difference analysis was conducted for experimental group A vs. control group B expert consensus. An independent samples *t*-test was adopted to test for differences between the two groups of students in creativity, aesthetic sensibility, and technical advantage. The results are as follows: creativity (*t* = 2.887; *p* < 0.05), technical advantage (*t* = 2.640; *p* < 0.05), aesthetic sensibility (*t* = 2.484; *p* < 0.05), indicating that there was a significant difference between the creative thinking teaching method and the traditional teaching method in the level of behavior (creativity, aesthetic sensibility, and technical advantage), as shown in Table 7.

**Table 7:** Independent sample *t*-test for two groups of creativity tendency consensus assessment.

**Items**	**Experimental group A**			**Control group B**			***t*-value**	***p*-value**
	**Mean**	**SD**	***N***	**Mean**	**SD**	***N***		
**Expert consensus**	4.2364	0.44224	23	3.8073	0.58579	20	2.732	0.009**
Creativity	4.3723	0.41989	23	3.9281	0.58499	20	2.887	0.006**
Aesthetic sensibility	4.1875	0.45812	23	3.7750	0.62743	20	2.484	0.017*
Technical advantage	4.1495	0.48219	23	3.7188	0.58753	20	2.640	0.012*

Mean is the average, SD is the standard derivation, and N is the number.

*p < 0.05.

**p < 0.01.

The full score of students' works is 100. The average score of expert consensus assessment is 85 in experimental group A (creative thinking teaching method) and 76 in control group B (traditional teaching method).

Six experts have given feedback on the learning outcomes of these two groups. Expert E1 said works from the experiment group were more complete and were finished quite better. Besides, Expert E2 said works from the experiment group were made of various materials and were of exhibition value. Importantly, Expert E4 said works from the experiment group were colorful and creative, making people full of imagination. Based on the feedback from experts, the control group attached more importance to details and skills, making works lack of creativity, said the expert E3. Besides, Expert E5 pointed out that works from the control group were made carefully and used a lot of skills taught by their teachers in the class. Furthermore, Expert E6 said both groups used methods of making lanterns taught by their teachers, but the experiment group was bolder and attracted my attention.

The works of the experimental group reflect the characteristics of bold colors, extended decoration, and dramatic emotional expression, echoing three important aspects of behavior. The expert evaluation results of the experimental group are better than the control group in creativity, aesthetic taste, and technical advantage. It can be found that the students who accept creative thinking teaching methods are better than those who accept traditional teaching methods in academic performance. Table 8 shows the classroom teaching situation of the experimental group and control group.

**Table 8:** Classroom teaching situation of experimental group A and control group B.

	**Experimental group A**	**Control group B**
Teaching situation	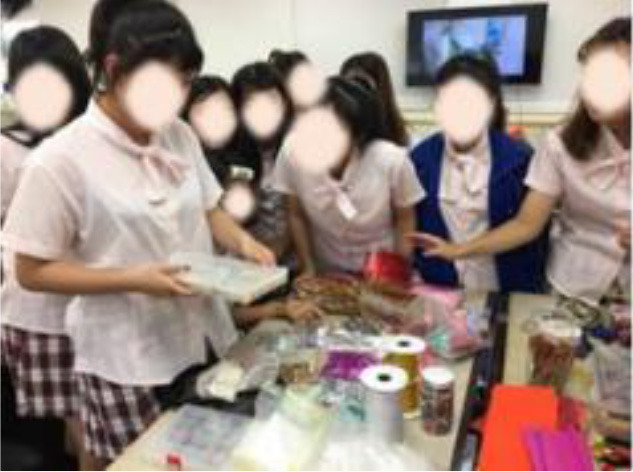	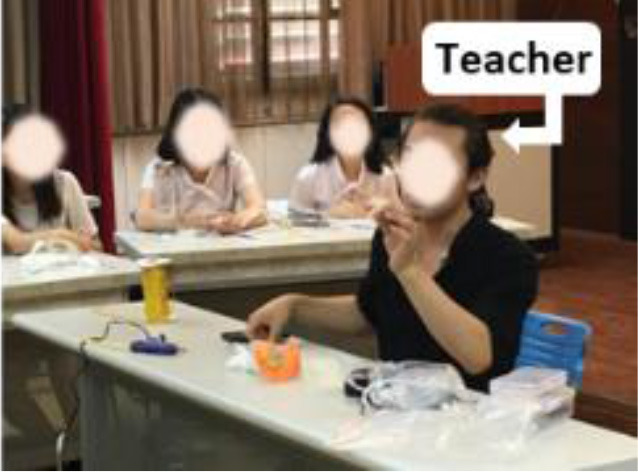
Learning situation	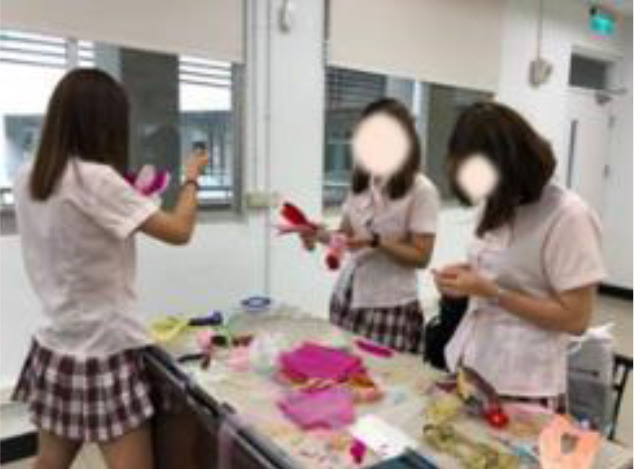	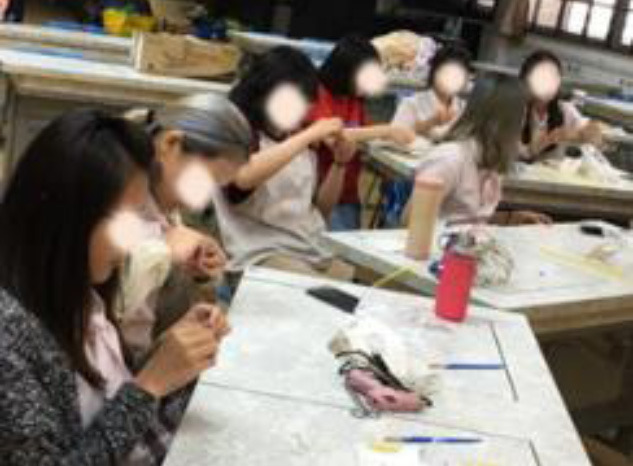
Final works	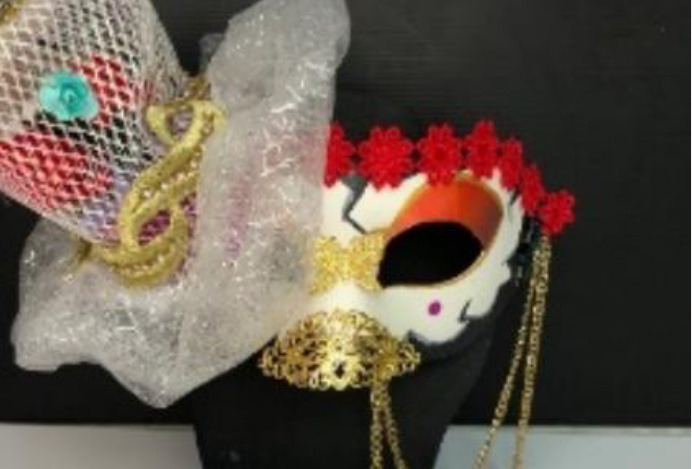	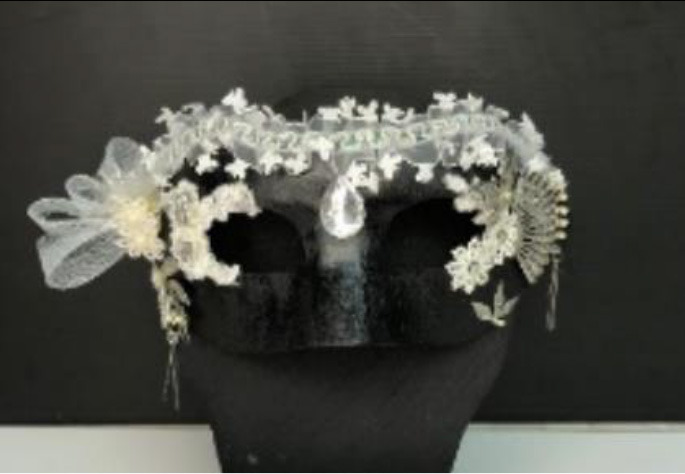

The results of expert consensus assessment show that creative thinking teaching method can effectively improve students' ability to conceive and create material works, in which the average value of creativity is the most significant, followed by technical advantages and aesthetic attraction, which is similar to that of Lai (2006) and Shih (2013).

**Figure 3:**
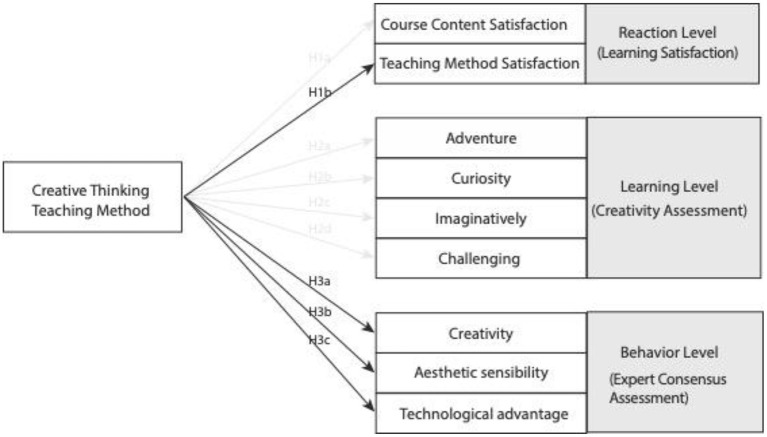
A summary of hypotheses with significant differences.

The hypotheses of H1b, h3a, H3b, and H3C are valid after being verified by statistical methods. Figure 3 shows the summary of the results of the hypothesis in this study.

## Discussion

Creativity is necessary for inventive thinking in any domain (Ocetkiewicz, 2021). However, is underappreciated in many Technical Education. This study integrates creative thinking teaching method into aesthetic beauty and hairdressing technical education courses. Students are divided into experimental group A and control group B to carry out creative thinking teaching methods and traditional teaching methods, respectively and make a comparative study on learning outcomes. The results show that there is a significant difference between the teaching method of creative thinking and the reaction level of learning outcomes. In the experimental group, the creative thinking teaching method is popular with students. These findings echoed the findings of previous studies of response level (Chang, 2004; Hung, 2004). Creative thinking can be applied to various disciplines, which can improve students' learning satisfaction (Li et al., 2011; Hu et al., 2016; Huang et al., 2021), also can enhance students' participation in the class (Tu et al., 2018). The creative thinking teaching method is positively correlated with creativity, aesthetic sensibility, and technical advantage. Therefore, the creative thinking teaching methods can effectively improve the learning effect. Additionally, creative thinking teaching method should contribute to the shifting away from traditional teaching, which on the learners as the broadcaster of content, toward a model based on functional learning.

In terms of teaching methods, the satisfaction with the creative thinking teaching method in the experimental group was higher than that of the traditional teaching method in the control group. The application of the brainstorming method, free association method, and six W's review method in the creative thinking teaching method can obviously stimulate students' more ideas and creativity, and effectively enhance their learning motivation, these findings are consistent with previous studies (Chen, 1999; Tan et al., 2016; Harris and de Bruin, 2018). As pointed out by Rodríguez et al. (2019) students rated their satisfaction with the course highly. That is to say, the creative thinking teaching method can effectively promote students' thinking and creativity, and the use of creative thinking teaching can help improve students' learning willingness and learning outcomes.

Although there's no significant difference between the creative thinking teaching methods and the creative tendency of learning outcomes, the creative thinking teaching methods hardly improve students' creative tendency. Even so, the scores of the creative tendency of the experimental group which applies the creative thinking teaching methods are better than that of the control group which applies the traditional teaching methods (Kennedy, 2010; Robson and Jaaniste, 2010; Davies et al., 2013). Among them, the difference in risk-taking level is the largest, the second is challenging, then are the imagination and curiosity, the latter of which the smallest. As is seen above, higher vocational students lack curiosity and imagination. In the future course arrangement, we suggest arranging more activities aimed to cultivate and stimulate students' curiosity and imagination. All teachers need to understand that creativity is a skill that may be developed in students (Ocetkiewicz, 2021). Accordingly, teachers must design strategies to support creative thinking teaching, thus encouraging and developing students' creativity.

According to expert consensus, creative thinking teaching methods can effectively improve students' ability to conceive multimedia material creations. The average of creativity improves the most and the next are the technical advantages and aesthetic sensibility, which is consistent with previous research results (Lai, 2006; Shih, 2013; Perry and Karpova, 2017). Knowing that every student can develop their creativity, teachers must formulate strategies to support creative thinking teaching, thus encouraging and fostering students' creativity.

## Conclusion

This study aims at exploring the effect of creative thinking teaching methods on multimedia material creation. It shows that it is feasible to introduce this method to beauty and hairdressing technical education. Creative thinking teaching methods can improve students learning motives, and inspire students' will to try different study methods, thus enhancing their creativity and learning outcomes. However, teachers should spend more time preparing for courses but feel happy about students' enthusiasm. To foster students' ESD, teachers should try more student-centered teaching methods to guide students' awareness and foster their creative thinking. The study, if taught through creative thinking teaching methods, is challenging for students, who will have a sense of achievement and be more willing to be involved. An increasing number of students believe that more creativity and creative ideas will be inspired when students consider how to solve problems, thus encouraging them to make more attempts and get a better learning outcome.

According to this research, creative teaching is a way that teachers inherit traditional teaching methods. With diversified teaching methods, it can inspire students' learning motivation and performance, increase their interest in teaching activities and encourage them to think to further foster their creative thinking. In the multimedia material creation course of beauty and hairdressing technical education, the creative thinking teaching methods can effectively improve students' learning outcomes, transform their thinking mode and break their thinking limits. As a result, students can put forward different ideas and answers, and make constant innovation, creating more creative and meaningful works. As society changes in the future, the traditional single thinking mode will not work, but creative thinking will contribute to students' sustainable learning. The creative thinking teaching method can make important contributions and is of significant value to teaching practice and academic research.

## Research limitations

This study also has some limitations. Firstly, the subjects of the study are 43 college students, which are limited in statistics. If the number of experimental samples can be increased, the inference statistics will be more accurate; Secondly, the gender distribution of the sample of research objects is mainly female (41 female students, accounting for 95%, 2 male students, accounting for 5%). The main reason is that the students of the department of applied cosmetology are mostly female, and the gender sample is very uneven, which makes the research results unable to verify whether the creative thinking teaching method is affected by gender. Secondly, Kirkpatrick proposed the four level training evaluations model (Kirkpatrick, 1975; Kirkpatrick and Kirkpatrick, 2006), because the “result level” is difficult to measure the time of learning outcomes, and it is not easy to determine the criteria for evaluating the results. Therefore, it is not included in the scope of the study. Future studies can include the “achievement level” to measure more accurate learning outcomes. Thirdly, due to the limitation of time, the research is limited to the differences in students' learning outcomes after the experimental course and fails to analyze the differences of students before and after the experimental course. Therefore, future research can further study the comparison between pre-test and post-test of experimental courses; Finally, if future researchers want to take college students as the research object, it is suggested to extend or increase the number of times in teaching time, expand the scope of teaching to other courses, and apply creative thinking teaching method more comprehensively, so as to promote students' creativity, so as to analyze whether students' creativity changes due to different teaching methods.

## Data availability statement

The original contributions presented in the study are included in the article/supplementary material, further inquiries can be directed to the corresponding authors.

## Ethics statement

Ethical review and approval was not required for the study on human participants in accordance with the local legislation and institutional requirements. The patients/participants provided their written informed consent to participate in this study. Written informed consent was obtained from the individual(s) for the publication of any potentially identifiable images or data included in this article.

## Author contributions

X-ZL and XK contributed to the ideas of creative thinking teaching research, collection of data, empirical analysis, the data analysis, design of research methods, and tables. C-CC and X-ZL participated in developing a research design, writing, and interpreting the analysis. X-ZL, C-CC, and XK contributed to the literature review and conclusions. All authors contributed to the article and approved the submitted version.

## Conflict of interest

The authors declare that the research was conducted in the absence of any commercial or financial relationships that could be construed as a potential conflict of interest.

## Publisher's note

All claims expressed in this article are solely those of the authors and do not necessarily represent those of their affiliated organizations, or those of the publisher, the editors and the reviewers. Any product that may be evaluated in this article, or claim that may be made by its manufacturer, is not guaranteed or endorsed by the publisher.
